# A Multi-Centre Randomized Study Comparing Two Standard of Care Chemotherapy Regimens for Lower-Risk HER2-Positive Breast Cancer

**DOI:** 10.3390/curroncol30080535

**Published:** 2023-08-04

**Authors:** Ricardo Fernandes, Terry L. Ng, Mashari Jemaan Alzahrani, Jacques Raphael, Phillip Blanchette, Morgan Black, Carol Stober, Gregory R. Pond, David Cella, Lisa Vandermeer, Mohammed Ibrahim, Mark Clemons

**Affiliations:** 1 Department of Oncology, Division of Medical Oncology, Schulich School of Medicine & Dentistry, Western University and London Health Sciences Centre, London, ON N6A 5W9, Canada; 2 Cancer Research Laboratory Program, Lawson Health Research Institute, London, ON N6C 2R5, Canada; 3 Department of Medicine, Division of Medical Oncology, The Ottawa Hospital and University of Ottawa, Ottawa, ON K1H 8L6, Canadamclemons@toh.ca (M.C.); 4 Cancer Therapeutics Program, Ottawa Hospital Research Institute, Ottawa, ON K1H 8L6, Canada; 5 Department of Oncology, McMaster University, Hamilton, ON L8V 5C2, Canada; 6 Department of Medical Social Sciences, Feinberg School of Medicine, Northwestern University, Chicago, IL 60611, USA; 7 Division of Clinical Sciences, Northern Ontario School of Medicine, Thunder Bay, ON P7B 5E1, Canada

**Keywords:** early-stage breast cancer, HER2-positive, trastuzumab, chemotherapy

## Abstract

Background: Neither paclitaxel plus trastuzumab (P-H) nor docetaxel-cyclophosphamide plus trastuzumab (TC-H) have been prospectively compared in HER2-positive early-stage breast cancer (EBC). A randomized trial was performed to assess the feasibility of a larger study. Methods: Lower-risk HER2-positive EBC patients were randomized to either P-H or TC-H treatment arms. The co-primary feasibility outcomes were: ≥75% patient acceptability rate, active trial participation of ≥50% of medical oncologists, ≥75% and ≥90% treatment completion, and receipt rate of planned cycles of chemotherapy, respectively. Secondary outcomes: Febrile neutropenia (FN) rate, treatment-related hospitalizations, health-related quality of life (HR-QoL) questionnaires. Analyses were performed by per protocol and intention-to-treat. Results: Between May 2019 and March 2021, 49 of 52 patients agreed to study participation (94% acceptability rate). Fifteen (65%) of 23 medical oncologists approached patients. Rates of FN were higher (8.3% vs. 0%) in the TC-H vs. P-H arm. Median (IQR) changes in scores from baseline in FACT-Taxane Trial Outcome Index at 24 weeks were −4 (−10, −1) vs. −6.5 (−15, −2) for TC-H and P-H arms, respectively. Conclusions: A randomized trial comparing P-H and TC-H was feasible. Expansion to a larger trial would be feasible to explore patient-reported outcomes of these adjuvant HER2 chemotherapy regimens.

## 1. Introduction

Excellent long-term outcomes of patients with human epidermal growth factor receptor 2 (HER2) positive early breast cancer (EBC) treated with trastuzumab-based treatments have been reported [1,2,3,4,5]. This means that performing trials comparing different durations of trastuzumab [2,6,7,8] and alternative chemotherapy backbones [3,4,9,10] has become challenging due to the large sample size and prolonged follow-up required. This has resulted in a move away from the traditional randomized study [11,12,13,14,15,16]. For example, the APT trial [3,17,18,19,20,21] evaluated the efficacy of weekly paclitaxel-trastuzumab in low-risk HER2-positive disease using a single-arm design of 410 patients and 1065 total patient-years of follow-up with comparison to historic controls. Similarly, a single-arm design is being applied in the ongoing DECRESCENDO trial that is evaluating pre-operative chemotherapy de-escalation and only giving more aggressive adjuvant chemotherapy in those patients who did not achieve a pathologic complete response [22]. The downside of these single-arm studies is that different standards of care will inevitably evolve, and comparative effectiveness with other regimens without a randomized comparator would be difficult [23]. For example, in the era of newer anti-HER2 therapies, such as in the phase II ATEMPT trial, although adjuvant trastuzumab emtansine (T-DM1) resulted in an excellent three-year invasive disease-free survival of 97.8%, this was not superior to paclitaxel/trastuzumab, and it did not reduce clinically relevant toxicity in patients with stage I HER2-positive breast cancer [24].

Despite their widespread use in lower-risk HER2-positive EBC, neither weekly paclitaxel plus trastuzumab (P-H) nor three-weekly docetaxel-cyclophosphamide plus trastuzumab (TC-H) have been prospectively compared [3,4,11,23]. This is despite the important differences between these chemotherapy regimens in terms of schedule (TC-H requires four three-weekly visits for TC and P-H requires 12 weekly visits for P), toxicity (three-weekly TC is associated with febrile neutropenia, weekly P is associated with increased peripheral neuropathy), and supportive care costs (TC frequently requires G-CSF support). While performing a definitive trial comparing different regimens using traditional invasive disease-free (iDFS) and overall (OS) survival endpoints would require a large sample size, the adoption of either regimen with no studies of comparative outcomes is clearly not optimal for patients, health care providers, or the health care system.

In the current study, we evaluated the feasibility of conducting a pragmatic clinical trial comparing these two regimens, using an Integrated Consent Model (ICM), in a population of patients with HER2-positive EBC. In addition, even if such a trial was feasible, it would require a large sample size, so we wished to assess other clinically relevant outcomes, such as toxicity, quality of life (QoL), patient-reported outcomes, and cost-effectiveness that could drive meaningful changes in clinical practice.

## 2. Methods

### 2.1. Study Design

A randomized, open-label feasibility trial performed across three Canadian cancer centres.

### 2.2. Patient Population

Potentially eligible patients were approached for study participation by their treating oncologist. Eligible criteria included: Histologically confirmed HER2-positive EBC for whom neo/adjuvant P-H or TC-H was being considered, no prior history of chemotherapy and ability to give oral consent as per the ICM [25,26]. Exclusion criteria included: Metastatic disease and patients unable to complete study questionnaires. Regulatory approval for this study was from the Western University Research Ethics Board (CTO1624).

### 2.3. Treatment and Assessments

Eligible and consented patients were randomized 1:1 to either:

Arm A: Weekly paclitaxel plus trastuzumab (P-H): Paclitaxel 80 mg/m^2^ weekly for 12 weeks and concurrently, trastuzumab 8 mg/kg followed by 6 mg/kg Day 1 every 21 days for 4 cycles, followed by trastuzumab 6 mg/kg Day 1 every 21 days to complete 1 year of trastuzumab therapy. Alternatively, participants could receive trastuzumab 4 mg/kg followed by 2 mg/kg weekly during the chemotherapy phase and then complete 1 year of trastuzumab.

Arm B: 3-weekly docetaxel-cyclophosphamide plus trastuzumab (TC-H): Docetaxel 75 mg/m^2^, Cyclophosphamide 600 mg/m^2^ and Trastuzumab 8 mg/kg followed by 6 mg/kg Day 1 every 21 days for 4 cycles, followed by trastuzumab 6 mg/kg Day 1 every 21 days to complete 1 year of Trastuzumab therapy.

Both regimens are provincially funded in Ontario, Canada and were given at standard approved doses and intervals. Assignment to the treatment groups was stratified by centre and neoadjuvant versus adjuvant treatment. Randomization used a permuted block design of 2 and 4. Allocation was performed either by the physician or by a research associate, using REDCap (Research Electronic Data Capture), a secure web-based software platform designed to support data collection developed by Vanderbilt University.

As this was a pragmatic trial, follow-up visits occurred as per usual care with no study-mandated schedules. Endpoint data were collected from several sources, including case report forms and from the patient’s electronic health records. Patient-reported outcomes were completed with self-completed questionnaires. The Functional Assessment of Cancer Therapy–Taxane Score (FACT-Taxane) (43 questions including the 27-item FACT-G and an 11-item score from a neurotoxicity subscale within a 16-item taxane subscale) is a validated measure of health-related QOL of patients receiving taxane-containing chemotherapy [27] and was measured at baseline, week 12 and 24. To avoid excessive use of patient questionnaires, an abbreviated version of the FACT-Taxane score (consisting of 10 questions: 4 on sensory neuropathy [NTX 1–4], 5 on taxane toxicity [TAX 1–5] and 1 item that queries bother with side effects, item GP5) was completed at weeks 3, 6, and 9 [27,28]. Functional Assessment of Chronic Illness Therapy (FACIT-Fatigue) is comprised of 13 questions subscales assessing fatigue experience and impact upon function. It is a validated measure of fatigue in people with cancer and other chronic health conditions [29,30] and was performed at baseline, weeks 3, 6, 9, 12, and 24. Edmonton Symptom Assessment System (ESAS) (10 questions) was collected at baseline and weeks 12 and 24 [31,32]. Health economic analyses were performed using FACT-8D 8-question cost utility questionnaire [33]. FACT-8D is validated for health system cost and cost per one quality-adjusted life year (QALY) gained and was collected at baseline and weeks 12 and 24 [27,30,32].

Adverse events were collected according to Common Terminology Criteria for Adverse Events (CTCAE) version 4.03 [34] at baseline, weeks 3, 6, 9, 12, and 24. Only specific adverse events of interest were collected (febrile neutropenia, grade 3 or 4 peripheral neuropathy, grade 3 or 4 cardiac toxicity, grade 3 or 4 diarrhea, grade 3 or 4 hematologic toxicity, grade 3 or 4 Taxane Acute Pain Syndrome (myalgias, arthralgias, musculoskeletal pain) and all events leading to hospitalization were also captured.

### 2.4. Objectives and Endpoints

The overall aim of the study was to assess the feasibility of conducting a pragmatic clinical trial using an Integrated Consent Model in a population of patients with HER2-positive EBC. If proven feasible, then a future large-scale clinical trial could be conducted comparing the potential efficacy of weekly P-H (paclitaxel plus trastuzumab) versus TC-H (docetaxel plus cyclophosphamide plus trastuzumab) in this patient population. We also wanted to preliminarily explore clinical safety and quality of life outcomes as secondary objectives.

Primary objective: To determine the feasibility of conducting a pragmatic clinical trial using an Integrated Consent Model with a combination of feasibility endpoints. The rates for each of these feasibility endpoints were established by discussion with study investigators before the study started and confirmed in the statistical analysis plan (SAP) (10 June 2020). For the study to be deemed feasible overall, the study must meet or exceed all of these criteria. These feasibility endpoints included:

The patient acceptability rate reflects the percentage of approached patients who were enrolled in the trial. To be deemed feasible this number must be ≥75%.

The active participation rate of medical oncologists is defined as the percentage of medical oncologists who approached patients for the study. To be deemed feasible, this number must be ≥50%.

The treatment receipt rate represents the percentage of enrolled patients who received their chemotherapy within the trial. To be deemed feasible, this number must be ≥90%.

The treatment completion rate is characterized by the percentage of expected chemotherapy cycles completed with no dose modifications (reductions, delays, or discontinuations). To be deemed feasible, this number must be ≥75%.

The recruitment rate is defined as the number of patients accrued to the study, divided by the duration of recruitment. To be deemed feasible, this number should be ≥2 patients/month.

The questionnaire completion rate represents the percentage of expected questionnaires that were completed per patient with no missing responses. To be deemed feasible, the questionnaire completion rate must be ≥80%.

### 2.5. Secondary Objectives

Toxicity rates using NCI CTC version 4.03 [34] reflect the frequency of toxicities, particularly febrile neutropenia, treatment-related hospitalizations, and peripheral neuropathy, in patients who received at least one dose of chemotherapy.

Frequency of febrile neutropenia prophylaxis in patients who received at least one dose of chemotherapy.

Quality of life outcomes were assessed using the FACT-Taxane, FACIT-Fatigue, and FACT-8D questionnaires. A 2-point difference in either the 4-item neurotoxicity subscale or the 5-item taxane subscale was felt a priori to be worth expanding this pilot study to a definitive study in a go/no-go sense.

### 2.6. Sample Size and Statistical Analysis

Baseline characteristics and all outcomes are presented descriptively, using means, medians, standard deviations, and ranges (continuous measures) or proportions (dichotomous or categorical data), as appropriate, along with 95% confidence intervals. Baseline characteristics and secondary outcomes were calculated separately for each treatment arm and calculated for both the ITT and PP populations. Given that the primary aim of this study was to evaluate study feasibility, safety outcomes are secondary aims. Therefore, any reporting of secondary outcomes prioritizes the PP population over the ITT population.

Differences in outcomes between treatment arms were estimated using descriptive statistics, along with 95% confidence intervals. The Kaplan–Meier method was used to estimate time-to-event outcomes. Statistical testing was performed using Fisher’s exact tests, Wilcoxon rank sum tests, or log-rank tests as appropriate. No adjustments were made for multiple tests, and exact *p*-values reported. All tests and confidence intervals are two-sided, and a *p*-value of 0.05 or less was considered statistically significant. However, appropriate caution was used in interpretation of statistically significant results. Inferences were performed understanding that efficacy and safety outcomes were considered secondary outcomes, and multiple tests were performed.

All the statistical analysis was carried out using SAS version-9 or higher for Windows (Cary, NC, USA) or R version 3.2.2. (www.r-project.org, accessed on 10 June 2020) or higher. The study was designed to recruit patients over a one-year period, during which it was estimated that 50 patients would be enrolled. This was deemed sufficient to answer the feasibility objectives of the study.

## 3. Results

The study opened at 3 Canadian cancer centres in Ontario, Canada (London, Ottawa, and Thunder Bay) on 1 May 2019, 19 August 2019 and 27 February 2020, respectively, and closed on 13 March 2021 at all sites. Between May 2019 and March 2021, 52 patients were approached to enter this study. Three patients declined the study. The most common reason was patient treatment preference. All remaining 49 patients were eligible and met all inclusion criteria.

**Figure 1:**
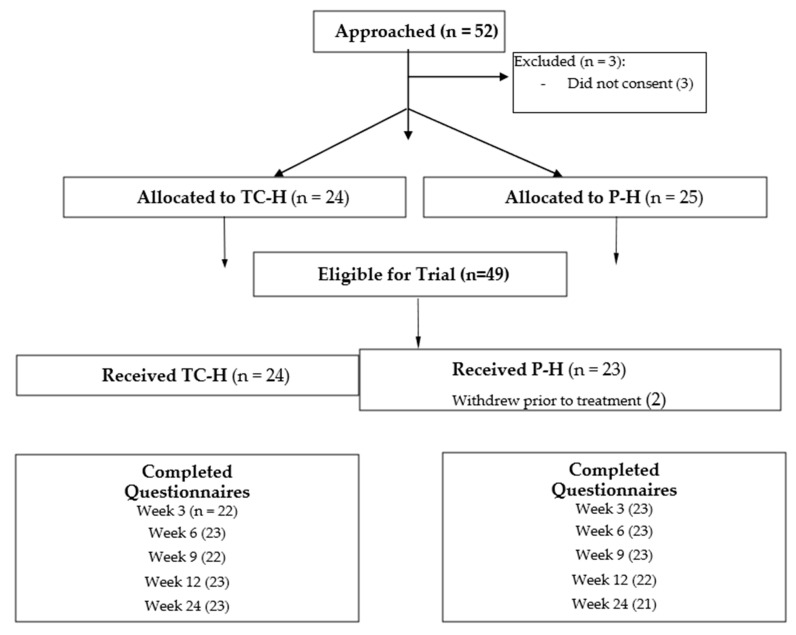
Trial Profile CONSORT 2010 Flow Diagram.

Of the 49 eligible patients, 25 (51%) were randomized to P-H and 24 (49%) to TC-H. The consort flow diagram depicting patient flow is shown in Figure 1. Patient baseline characteristics and descriptive statistics for the entire cohort are summarized in Table 1. The median age was 61 years (range 23–79). Median tumor size was 2 cm (0.7–7) and 8 patients (16.3%) had N1 disease. Regarding cancer treatment, the majority (38/49, 78%) received it in the adjuvant setting (Table 1). Of 23 physicians who signed study logs, 15 (65%) approached patients. By individual study site, this ratio was: London (7/10, 70%), Ottawa (7/8, 88%), and Thunder Bay (1/5, 20%).

Of 49 patients who agreed to participate in the trial and were randomized, 47 (95%) received their treatment as per protocol. As the study ran for 23, 20, and 14 months at the London, Ottawa, and Thunder Bay sites, respectively, the overall duration of the study was 23 months. Therefore, the recruitment rate was 2.13 patients per month.

Treatment information is provided in Table 2. Of the 49 patients who were randomized and started on chemotherapy, 38 (77.6%) completed all planned cycles of chemotherapy without any dose delays or reductions. By chemotherapy arm, while all twenty-four (100%) patients completed TC-H, 2/25 patients allocated to receive P-H withdrew from the study because they did not want to complete the QOLs, thus excluded from further analyses. Median time to starting chemotherapy from randomization was 12 and 15 days, respectively, for patients receiving TC-H and P-H.

### 3.1. Secondary Endpoints

The rates of toxicity for the two study arms are summarized in Table 2. The rates of FN were higher in the TC-H arm vs. the P-H arm (8.3% vs. 0%). Of note, 66% (16/24) of participants in the TC-H arm received primary febrile neutropenia prophylaxis while 4% (1/24) received secondary. Treatment-related hospitalization was more common with TC-H arm (2/24; 8.3%) than with P-H arm (1/25; 4.4%). One patient on P-H arm had a port-a-cath infection.

Eight patients in each arm had a dose reduction (33.3% TC-H versus 34.8% P-H arms). However, only one patient (4.2%) on TC-H prematurely discontinued chemotherapy compared with 7 (30.4%) patients on P-H. While the most common reason for dose reduction was diarrhea (2 patients in each arm), peripheral neuropathy led to chemotherapy discontinuation in 20% of the patients in the TC-H arm. Two patients on the P-H arm had a change in their chemotherapy regimen—one patient switched to abraxane due to a reaction to paclitaxel, and another patient added carboplatin.

### 3.2. Quality of Life Endpoints

ESAS scores as measured at baseline, week 12 and week 24 are provided in Table 3. The absolute FACT questionnaire scores are presented in Table 4, while the changes in FACT scores from baseline are in Table 5. For the 49 patients who entered the study, the number who completed all their study questionnaires was 44 (89%).

Following the 12 weeks of chemotherapy, patients treated with TC-H had significantly lower median (IQR) changes in scores (Table 5) on the FACT-F in comparison to P-H (−9 (−15, −3) versus −3.5 (−14, 2), respectively), favoring P-H regimen. Regarding TC-H arm, the FACT-G and FACT-8D questionnaires had lower median (IQR) changes scores at 12 weeks of chemotherapy (−7 (−12, 1) and −6 (−12, 3), respectively). Median (IQR) changes in scores from baseline in FACT-Taxane Trial Outcome Index at 24 weeks were −4 (−10, −1) vs. −6.5 (−15, −2) for TC-H and P-H arms, respectively.

## 4. Discussion

Both TC-H (4 cycles of 3-weekly docetaxel (T) and cyclophosphamide (C) with 1 year of 3-weekly trastuzumab (H)), and P-H (12 weeks of weekly paclitaxel (P) and 1 year of 3-weekly trastuzumab (H)) are widely used in patients with lower-risk HER2-positive EBC [16,23]. However, despite the differences between these chemotherapy regimens in terms of schedule, toxicity, and supportive care costs, these regimens have never been compared in a head-to-head randomized trial. The available data for their efficacy relative to other chemotherapy regimens are, therefore, limited [16,23]. To our knowledge, this is the first prospective randomized trial comparing them.

The primary objective of this study was to evaluate whether performing a future confirmatory trial with DFS or OS as the primary outcome would be feasible. The study confirmed the feasibility of doing such a trial, with 65% of oncologists approaching patients and 95% of patients received their treatment as per the arm of the study to which they were randomized. Unfortunately, to detect even a modest effect (e.g., hazard ratio of 0.80), assuming a 2-year DFS of 95% would require in excess of 5000 patients accrued and a study duration of at least 7 years. Hence, even though the majority of feasibility endpoints were met, this does not remove the barriers to performing such a trial. Use of other endpoints, such as toxicity or QOL, could allow a smaller and shorter study to be performed. Based on the data observed in this study, TC-H was associated with detrimental scores on FACT-T, FACT-G and FACT-F questionnaires, while early discontinuation of chemotherapy was more common in the P-H arm.

The limitations of this study are recognized and include a small sample size, open-label design, and participation of patients from a single country. Patients had to complete multiple questionnaires during the study, which was arduous and time-consuming. It also restricts the study sample size to modest number of participants to keep the study feasible. It is also important to mention that the patient population did include some high-risk characteristics, such as node-positive disease and larger tumors. Overall, 16.8% of patients had node-positive disease and 1 patient had a 7 cm breast tumor. The increased rate of FN observed in the TC arm could also reflect the lower use of primary neutropenia prophylaxis than one would normally expect. It is, therefore, possible that great use of prophylaxis could have reduced the QOL differences between the two arms. Finally, as this was a feasibility trial, there will be a bias in accrual towards those physicians who truly believe in the study. That being said, 65% of medical oncologists among the three study sites who treat breast cancer, agreed to study participation, therefore, it reflects a high acceptance of the study proposal.

In conclusion, the importance of comparing chemotherapy regimens in a real-world population with either lower-risk breast cancer or increased frailty, assessing the relative benefits and toxicity of a chosen chemotherapy regimen is essential, particularly in patients with low-risk HER2-positive disease, which are underrepresented in the pivotal randomized adjuvant trials [14,15]. While we have demonstrated the feasibility of accrual, it is apparent that performing a larger efficacy trial will not be possible in the current funding environment and would require thousands of patients and extended follow-up. On the other hand, we have also shown that the use of other endpoints, such as toxicities and quality of life, such FACT-T and FACT-G, may be important to allow such a study to be performed with a smaller sample size and shorter follow-up.

## Figures and Tables

**Table 1:** Baseline Characteristics.

Patients Approached		All Patients (N = 52)
Eligibility Criteria		
	London	14 (26.9)
Study Centre	Ottawa	37 (71.2)
	Thunder Bay	1 (1.9)
Timing of Chemotherapy	Neoadjuvant	11 (21.2)
	Adjuvant	41 (78.9)
Age ≥ 18 with HER2-positive early-stage Breast CA	N (%) Yes	52 (100)
Able to Provide Verbal Consent	N (%) Yes	52 (100)
Willing to Complete Study Questionnaires	N (%) Yes	52 (100)
Metastatic Breast CA	N (%) Yes	0 (0)
Provided Consent	N (%) Yes	49 (94.2)
**Patients Enrolled (N = 49)**		
Demographics		
	London	14 (28.6)
Study Centre	Ottawa	34 (69.4)
	Thunder Bay	1 (2.0)
Timing of Chemotherapy	Neoadjuvant	11 (22.5)
	Adjuvant	38 (77.6)
Randomization Group	A: TC-H	24 (49.0)
	B: P-H	25 (51.0)
Age at Study Registration	Mean (sd)	58.8 (11.9)
	Median (range)	61 (23, 79)
Gender	N (%) Female	49 (100.0)
Cancer		
HER2 Status	N (%) Positive	49 (100)
N Stage	N (%) 0	41 (83.7)
	1	8 (16.3)
M Stage	N (%) 0	49 (100)
Size	Median (range)	2 (0.7, 7.0)

**Table 2:** Treatments.

Treatments			
		TC-H	P-H
N		24	25
Off-Study Reason	Completed Study	24 (100)	23 (92.0)
	Withdrew	0 (0)	2 (8.0)
N		24	23
Days to Chemotherapy	Median (range)	12 (4, 23)	15 (1, 36)
Duration of Chemotherapy	Median (range)	63 (42, 69)	77 (0, 85)
Docetaxel Dose	Median (range)	300 (225, 300)	-
Cyclophosphamide Dose	Median (range)	2400 (1800, 2400)	-
Paclitaxel Dose	Median (range)	-	880 (80, 1200)
Chemotherapy Changes	N (%) Yes	0	2 (8.7)
	Switched to abraxane	0	1
	Added carboplatin	0	1
Treatment Delays	N (%) Yes	1 (4.2)	5 (21.7)
	ANC	0	1
	Port-a-cath infection	0	2
	Bony pain, diarrhea, abdominal discomfort	1	0
	Neuropathy	0	1
	Diarrhea, fatigue, vaginal/hem bleed	0	1
Dose Reductions	N (%) Yes	8 (33.3)	8 (34.8)
	Pain	1	0
	Diarrhea	2	2
	Gr 3 neutropenia	0	1
	FN	1	0
	Liver enzymes	0	1
	Elevated ALT	0	1
	Fatigue	1	1
	Age	0	1
	Macular popular rash	1	0
	Mucositis/leg discomfort	1	0
	Neuropathy	1	1
	Tolerability	1	0
Chemotherapy Discontinued	N (%) Yes	1 (4.2)	7 (30.4)
	Allergic reaction	0	2
	Staph aureus port infection	0	1
	Metastatic disease	1	0
	Neuropathy	0	2
	Peripheral neuropathy	0	1
	Pt choice	0	1
Treatment-Related Hospitalization	N (%) Yes	2 (8.3)	1 (4.4)
	FN	2	0
	Port-a-cath Infection	0	1
Days On-Study	Median (range)	180 (116, 275)	181 (77, 203)
Serious Adverse Events	N (%) Yes	2 (8.3)	1 (4.4)
	FN	2	0
	Port-a-cath Infection	0	1

ANC: Absolute neutrophil count; FN: Febrile neutropenia; ALT: Alanine transaminase.

**Table 3:** Median (IQR) ESAS Scores.

ESAS Domain	TC-H	P-H
Baseline		
Pain	2 (0, 4)	1 (0, 2)
Tiredness	1 (0, 5)	2 (1, 4)
Drowsiness	0 (0, 4)	0 (0, 2)
Nausea	0 (0, 0)	0 (0, 0)
Loss of Appetite	0 (0, 2)	0 (0, 0)
Shortness of Breath	0 (0, 0)	0 (0, 0)
Depression	0 (0, 2)	1 (0, 3)
Anxiety	2 (0, 4)	3 (1, 5)
Well-Being	2 (0, 3)	2 (1, 3)
Other Problem	0 (0, 1)	0 (0, 0)
Week 12		
Pain	0 (0, 2)	1 (0, 4)
Tiredness	4 (1, 6)	4 (2, 7)
Drowsiness	2 (0, 5)	2 (0, 4)
Nausea	0 (0, 0)	0 (0, 0)
Loss of Appetite	0 (0, 3)	0 (0, 1)
Shortness of Breath	0 (0, 2)	0 (0, 1)
Depression	2 (0, 3)	2 (0, 3)
Anxiety	1 (0, 3)	2 (0, 3)
Well-Being	3 (1, 4)	3 (1, 4)
Other Problem	0 (0, 0)	0 (0, 3)
Week 24		
Pain	1 (0, 3)	2 (0, 4)
Tiredness	3 (1, 5)	2 (2, 4)
Drowsiness	1 (0, 3)	1 (0, 2)
Nausea	0 (0, 0)	0 (0, 0)
Loss of Appetite	0 (0, 0)	0 (0, 1)
Shortness of Breath	0 (0, 1)	0 (0, 1)
Depression	0 (0, 3)	2 (0, 4)
Anxiety	1 (0, 3)	2 (0, 6)
Well-Being	2 (1, 3)	3 (1, 5)
Other Problem	0 (0, 2)	0 (0, 0)

**Table 4:** Median (IQR) FACT Scores.

FACT	TC-H	P-H
Baseline		
FACT-F	47 (41, 49)	44 (42, 48)
FACT-8D	0.53 (0.39, 0.56)	0.47 (0.40, 0.55)
Physical Well-Being	26 (23, 27)	26 (22, 27)
Functional Well-Being	24 (15, 28)	20 (19, 23)
Emotional Well-Being	19 (16, 21)	17 (16, 20)
Social Well-Being	26 (20, 28)	21 (19, 23)
Taxane Score	58 (52, 63)	60 (57, 62)
Taxane TOI	107 (7, 114)	105 (96, 110)
FACT-G	94 (74, 100)	85 (77, 90)
Taxane Total Score	151 (136, 161)	145 (133, 150)
Week 3		
FACT-F	40 (34, 48)	44 (35, 47)
Abbreviated Neurotoxicity Score	16 (14, 16)	16 (15, 16)
Abbreviated Taxane Score	20 (17, 20)	19 (17, 20)
Week 6		
FACT-F	41 (28, 45)	41 (31, 46)
Abbreviated Neurotoxicity Score	16 (14, 16)	16 (13, 16)
Abbreviated Taxane Score	18 (17, 20)	19 (17, 20)
Week 9		
FACT-F	37 (24, 43)	34 (29, 44)
Abbreviated Neurotoxicity Score	15 (13, 16)	14 (12, 16)
Abbreviated Taxane Score	18 (16, 20)	19 (16, 20)
Week 12		
FACT-F	37 (28, 44)	36.5 (27, 46)
FACT-8D	0.54 (0.43, 0.65)	0.47 (0.39, 0.63)
Physical Well-Being	23 (19, 25)	22 (17, 24)
Functional Well-Being	18 (12, 23)	18 (12, 21)
Emotional Well-Being	19 (18, 21)	20 (17, 22)
Social Well-Being	23 (18, 25)	22 (19, 24)
Taxane Score	55 (49, 61)	53 (47, 60)
Taxane TOI	93 (80, 105)	95 (74, 104)
FACT-G	82 (67, 92)	76 (64, 86)
Taxane Total Score	133 (119, 150)	135 (114, 141)
Week 24		
FACT-F	43 (36, 47)	40 (36, 46)
FACT-8D	0.53 (0.47, 0.60)	0.53 (0.41, 0.58)
Physical Well-Being	25 (23, 27)	23 (20, 26)
Functional Well-Being	20 (14, 23)	18 (12, 22.5)
Emotional Well-Being	20 (19, 23)	20 (17, 23)
Social Well-Being	23 (17, 27)	20 (16, 25)
Taxane Score	57 (50, 61)	60 (51, 61)
Taxane TOI	99 (90, 107)	100 (82, 103.5)
FACT-G	87 (74, 96)	78 (67, 90)
Taxane Total Score	143 (131, 152)	136 (115, 149.5)

**Table 5:** Median (IQR) Change in Scores from Baseline.

FACT	TC-H	P-H
Baseline		
FACT-F		
FACT-8D		
Physical Well-Being		
Functional Well-Being		
Emotional Well-Being		
Social Well-Being		
Taxane Score		
Taxane TOI		
FACT-G		
Taxane Total Score		
Week 3		
FACT-F	−4 (−10, −1)	−2 (−5, 4)
Week 6		
FACT-F	−4 (−9, −1)	−2 (−9, 3)
Week 9		
FACT-F	−7.5 (−20, −3)	−6 (−18, 0)
Week 12		
FACT-F	−9 (−15, −3)	−3.5 (−14, 2)
FACT-8D	0.05 (−0.07, 0.18)	−0.01 (−0.13, 0.09)
Physical Well-Being	−2 (−4, 0)	−3.5 (−8, 1)
Functional Well-Being	−3 (−8, −1)	−2 (−5, 0)
Emotional Well-Being	1 (−2, 4)	2 (1, 3)
Social Well-Being	−2 (−4, 1)	0 (−3, 4)
Taxane Score	−2 (−9, 3)	−4 (−9, 0)
Taxane TOI	−9 (−14, −3)	−9.5 (−23, −3)
FACT-G	−7 (−12, 1)	−6 (−12, 3)
Taxane Total Score	−9 (−18, 5)	−7 (−21, −2)
Week 24		
FACT-F	−3 (−6, −1)	−4 (−10, 1)
FACT-8D	0.02 (−0.03, 0.14)	0.02 (−0.05, 0.11)
Physical Well-Being	0 (−2, 0)	−1 (−6, 0)
Functional Well-Being	−2 (−5, 1)	−2 (−5.5, 2)
Emotional Well-Being	2 (0, 4)	2 (1, 4)
Social Well-Being	−1 (−3, 2)	0 (−5, 2)
Taxane Score	−2 (−5, 3)	−2 (−7, 0)
Taxane TOI	−4 (−10, −1)	−6.5 (−15, 2)
FACT-G	−1 (−9, 2)	−4 (−13.5, 2.5)
Taxane Total Score	−3 (−12, 6)	−7 (−20, 8.5)

## Data Availability

De-identified participant data sharing will be available after publication with appropriate data access agreement.
